# In-Hospital Deaths From Ambulatory Care–Sensitive Conditions Before and During the COVID-19 Pandemic in Japan

**DOI:** 10.1001/jamanetworkopen.2023.19583

**Published:** 2023-06-22

**Authors:** Kazuhiro Abe, Ichiro Kawachi, Arisa Iba, Atsushi Miyawaki

**Affiliations:** 1 Takemi Program in International Health, Harvard T.H. Chan School of Public Health, Boston, Massachusetts; 2 Department of Public Health, Graduate School of Medicine, The University of Tokyo, Tokyo, Japan; 3 Department of Social and Behavioral Sciences, Harvard T.H. Chan School of Public Health, Boston, Massachusetts; 4 Institute for Global Health Policy Research, Bureau of International Health Cooperation, National Center for Global Health and Medicine, Tokyo, Japan

## Abstract

**Importance:**

The COVID-19 pandemic may have played a role in the deterioration of access to medical care for ambulatory care–sensitive conditions (ACSCs).

**Objective:**

To ascertain whether the number of in-hospital deaths and in-hospital mortality rate associated with ACSC changed after the declaration of the COVID-19 national state of emergency in Japan.

**Design, Setting, and Participants:**

This cohort study used a difference-in-differences design to compare outcomes for ACSC in the period before (January 1, 2015, to December 31, 2019) vs the period after (January 1, 2020, to December 31, 2020) Japan declared a national state of emergency for the COVID-19 pandemic. Analyses used discharge summary data from 242 acute care hospitals across Japan. The sample comprised unscheduled hospitalizations of patients with ACSC during the study period (January 1, 2015, to December 31, 2020). Data analyses were performed between August 16 and December 7, 2022.

**Exposure:**

The declaration of the COVID-19 national state of emergency announced by the Japanese government in April 2020 was considered to be an exogenous shock.

**Main Outcomes and Measures:**

Primary outcomes were the ACSC-associated number of in-hospital deaths, number of hospitalizations, and in-hospital mortality rate.

**Results:**

A total of 28 321 ACSC-related hospitalizations were observed involving 15 318 males (54.1%), with a median (IQR) age of 76 (58-85) years. The number of in-hospital deaths was 2117 (7.5%). The number of hospitalizations decreased overall (incidence rate ratio [IRR], 0.84; 95% CI, 0.75-0.94), for chronic conditions (IRR, 0.84; 95% CI, 0.77-0.92), and for vaccine-preventable conditions (IRR, 0.58; 95% CI, 0.44-0.76). However, in-hospital deaths (IRR, 1.66; 95% CI, 1.15-2.39) and in-hospital deaths within 24 hours of hospital arrival (IRR, 7.27 × 10^6^; 95% CI, 1.83 × 10^6^ to 2.89 × 10^7^) increased for acute conditions. The in-hospital mortality rate increased for acute conditions (IRR, 1.71; 95% CI, 1.16-2.54), and the 24-hour in-hospital mortality rates also increased overall (IRR, 1.87; 95% CI, 1.19-2.96), for acute conditions (IRR, 2.15 × 10^6^; 95% CI, 5.25 × 10^5^ to 8.79 × 10^6^), and for vaccine-preventable conditions (IRR, 4.64; 95% CI, 1.28-16.77).

**Conclusions and Relevance:**

This cohort study found that in Japan, the number of in-hospital deaths increased after the declaration of the COVID-19 national state of emergency in 2020, particularly for acute ACSC and deaths within 24 hours of hospital admission. This finding suggests that access to good-quality primary care and inpatient care for patients with acute ACSC may have been compromised during the pandemic.

## Key Points

**Question:**

Did patients with ambulatory care–sensitive conditions (ACSCs) experience worse outcomes in hospitals during the COVID-19 pandemic in Japan?

**Findings:**

In this cohort study of 28 321 ACSC-associated hospitalizations, in-hospital mortality rates within 24 hours of hospital arrival increased overall, for acute ACSC (eg, dehydration and gastroenteritis), and for vaccine-preventable conditions (eg, bacterial pneumonia). The increased mortality was associated with not only a decrease in the number of hospitalizations but also an increase in in-hospital deaths.

**Meaning:**

Findings of this study suggest that patients with ACSC experienced worse outcomes during the pandemic, suggesting that access to good-quality primary care and inpatient care could have been compromised.

## Teaser

This cohort study compares prepandemic with pandemic mortality and hospitalization data among patients with ambulatory care–sensitive conditions.

## Introduction

The COVID-19 pandemic led to substantial changes in the behavior of patients and health care practitioners across many countries. Earlier in the pandemic, many patients were dissuaded from seeking health care due to fear of being infected in clinical settings inundated by patients with COVID-19.^1,2^ A decrease in outpatient care, including vaccinations, cancer screenings, and sexually transmitted disease testing, was reported during the pandemic.^3,4,5,6,7,8^ At the same time, waiting times for admissions and elective surgeries increased due to overburdened medical institutions.^9,10,11^ Given that continuity of patient access to regular visits, preventive care, and inpatient care is an essential element for ensuring the quality of primary care, there is concern that the pandemic played a role in the deterioration of access to high-quality primary care.

The standardized number of hospitalizations due to ambulatory care–sensitive conditions (ACSCs) is a widely used indicator to monitor the quality of primary care worldwide. Ambulatory care–sensitive condition is defined as a preventable condition in primary care (eg, dehydration and gastroenteritis, asthma, congestive heart failure, and pneumococcal pneumonia).^12,13^ Previous studies in Canada and the US found that the number of hospitalizations and emergency department visits due to ACSC decreased early in the pandemic,^14,15^ whereas intensive care unit admission rates and length of stay remained unchanged.^15^ Even in Japan, where the number of patients with COVID-19 was lower than in Canada and the US,^16^ a decrease in the number of ACSC-related hospitalizations has been reported.^17^ However, it can be difficult to interpret these findings based on indicators alone, such as the number of hospital admissions.^15^ For example, it is not clear whether the decrease in ACSC-related hospitalizations during the pandemic was a good outcome, as it is possible that patients who needed to be admitted likely simply could not gain entry into hospitals. Hence, it is necessary to examine changes in outcomes for patients with ACSC during the pandemic.

Accordingly, this study sought to determine whether the number of in-hospital deaths and in-hospital mortality rate associated with ACSC increased after the declaration of the COVID-19 national state of emergency in Japan. We used a difference-in-differences (DID) framework based on discharge summary data from 242 Japanese acute care hospitals. Process indicators, such as the number of hospitalizations, length of stay, and ambulance transport rates, were also compared with the findings of previous studies. In addition, a triple-difference approach was used to analyze changes in outcomes according to hospital size.

## Methods

### Study Design, Participants, and Data Sources

We performed a DID analysis, comparing the difference in mean outcomes between the months before vs after the COVID-19 national state of emergency declaration (January 1, 2020, to December 31, 2020) and the corresponding months prior to the pandemic (January 1, 2015, to December 31, 2019).^18^ The declaration of a national state of emergency announced by the Japanese government in April 2020 was considered to be an exogenous shock. It encouraged the public to refrain from going out and gathering, to work remotely, and to prevent the spread of infection by masking and handwashing.^16^ The University of Tokyo Ethics Review Committee approved this study and waived the informed consent requirement because we used deidentified data. We followed the Strengthening the Reporting of Observational Studies in Epidemiology (STROBE) reporting guideline.

The analytic sample comprised unscheduled hospitalizations of patients with ACSC during the study period. Scheduled hospitalizations and hospitalizations of neonates born at those hospitals were excluded from the analysis. Information on patients with ACSC was extracted from discharge summary data completed by the responsible physician. Study data were obtained from a deidentified hospital claims database of 242 acute care hospitals between January 1, 2015, and December 31, 2020, compiled by Medical Data Vision Co Ltd.^19,20^ The data included 11% of all hospital admissions in Japan and had distributions of patient age, sex, and principal diagnosis that were similar to the nationwide estimates according to the patient survey conducted by the Ministry of Health, Labour and Welfare of Japan.^20^

We used a 5% random sample data of patients with a visit history in these acute care hospitals during the study period. The data included the diagnosis for each patient based on the *International Statistical Classification of Diseases and Related Health Problems, Tenth Revision (ICD-10)* diagnosis codes; patient age at admission, sex, route of hospitalization (ie, transport by ambulance vs other), in-hospital death information (ie, in-hospital death within 24 hours of hospital arrival, in-hospital death, or other), and length of stay; and hospital capacity (ie, <200 beds vs ≥200 beds). Individual data were then aggregated on a monthly basis.

### Definition of ACSC

In this study, we used the definition of ACSC from the UK National Health Service^12^ because previous studies in Japan have used it and because Japan has not yet established its own ACSC definition.^17,21^ The ACSCs were categorized as acute, including 9 conditions (eg, dehydration and gastroenteritis); chronic, including 8 conditions (eg, asthma and congestive heart failure); or vaccine-preventable, including 2 conditions (eg, pneumococcal pneumonia). Disease definitions and their corresponding *ICD-10* codes are provided in eTable 1 in Supplement 1.

The discharge summary data included several *ICD-10* diagnosis codes: admission-precipitating diagnosis, primary diagnoses, conditions for which the most medical resources were invested, and comorbidities at hospitalization.^21^ We identified the ACSC diagnosis for hospitalization from the admission-precipitating diagnosis. If this admission-precipitating diagnosis was not recorded, the primary diagnoses were used and then the conditions for which the most medical resources were expended.^21^ Of all of the hospitalizations during the study period, 3 instances had no admission-precipitating diagnosis, but all patients admitted with ACSC had an admission-precipitating diagnosis.

### Outcomes and Covariates

The primary outcomes were the number of in-hospital deaths, the number of hospitalizations, and the in-hospital mortality rate associated with ACSC. The number of in-hospital deaths was separated into deaths within 24 hours vs deaths after 24 hours of hospital arrival. The in-hospital mortality rate was defined as the number of deaths divided by the number of hospitalizations per month, and the in-hospital mortality rate within 24 hours was defined as the number of deaths within 24 hours of hospital arrival divided by the number of hospitalizations per month. The denominator and numerator of each ACSC were used to calculate the respective mortality rates.

Secondary outcomes were the mean length of stay and ambulance transport ratio. The ambulance transport ratio was the number of patients who were transported to the hospital by ambulance as a proportion of total hospitalizations per month. For each outcome, we considered ACSCs overall and as acute, chronic, or vaccine-preventable. As a point of comparison, we examined outcomes of hospitalization for patients who were admitted with acute myocardial infarction (AMI), for which inpatient treatment is essential and for which there has been research on patient outcomes during the pandemic in Japan.^22^

The median age of patients, percentage of female patients, and Elixhauser comorbidity index (calculated from *ICD-10* codes indicating complications at hospitalization; range: 0-30, with the highest scores indicating multiple comorbidities) were added in sensitivity analysis to account for potential confounding by changes in the severity of the condition for hospitalized patients.^23,24^

### Statistical Analysis

We performed a DID analysis using Poisson regression with robust SEs to estimate the statistical significance of the association.^18^ In equation 1: 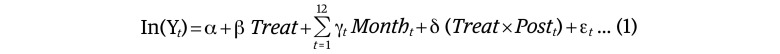, where γ_t_ was the outcome variables at month t, *Treat* was the indicator for the treatment period (ie, pandemic period [2020] vs prepandemic period [2015- 2019]), *Month_t_* was the dummy variable at month t, and *Post_t_* indicated 1 if the month *t* was April or later and indicated 0 if otherwise. α, β, γ, and δ were coefficients, where δ was the causal parameter of interest; ε was the error term. Incidence rate ratios (IRRs) were calculated as exp(δ).

In addition, to confirm the trend in monthly IRRs, we used equation 2: 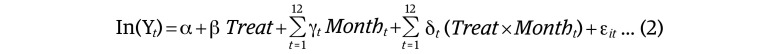, which was estimated by Poisson regression using robust estimators. We plotted the IRRs for each month calculated from δt.

Moreover, we adopted a triple-difference approach to estimate the differences in the change in each outcome by hospital size, and equation 3 was estimated using a modified Poisson regression analysis.

ln (Y*_t_*) = α +β *Treat* + γ*Post_t_* +ρ*Capacity* + σ(*Treat* × *Post_t_*) + τ(*Post_t_* × *Capacity*) + 

φ(*Capacity* × *Treat*) + δ(*Treat* × *Post_t_* × *Capacity*) + ε_t_ (3), where α, β, γ, ρ, σ, τ, φ, and δ were coefficients, and δ was the causal parameter of interest. Capacity indicated 1 for hospitals with more than 200 beds and indicated 0 if otherwise.

For the sensitivity analysis, we performed regression, adjusting for additional potential confounders in equations 1 and 3. All analyses were conducted between August 16 and December 7, 2022, using Stata, version 16 MP (StataCorp LLC). A 2-sided *P* < .05 was interpreted as statistically significant.

## Results

A total of 28 321 ACSC-related hospitalizations were included in the analysis, involving 13 003 females (45.9%) and 15 318 males (54.1%), with a median (IQR) age of 76 (58-85) years. Of these hospitalizations, 24 261 occurred during the prepandemic period and 4060 during the pandemic period. Total hospitalizations consisted of 7301 admissions (25.8%) for acute conditions, 17 015 admissions (60.1%) for chronic conditions, and 4005 admissions (14.1%) for vaccine-preventable conditions. The number of in-hospital deaths was 2117 (7.5%).

The mean (SD) number of hospitalizations decreased in the pandemic period compared with the prepandemic period (eg, ACSC, April-December: 313.3 [38.1] vs 393.9 [35.5]) (Table 1). Comparing prepandemic with pandemic in-hospital deaths, the mean (SD) number of deaths either decreased or remained unchanged for all categories (eg, ACSC, April-December: 28.0 [6.5] vs 24.8 [3.9]). On the other hand, the mean (SD) number of in-hospital deaths within 24 hours of hospital arrival increased in the pandemic period after April compared with the prepandemic period (eg, ACSC, April-December: 5.4 [3.6] vs 3.6 [1.9]). Moreover, ACSC-related hospitalizations after April 2020 (the pandemic period) were characterized by older age, higher Elixhauser comorbidity index, shorter length of stay, and a higher rate of ambulance transport (eTable 2 in Supplement 1).

**Table 1.** Monthly Statistics of Hospitalizations Due to ACSCs in Prepandemic and Pandemic Periods

Outcome variable	Period, mean (SD)			
	Prepandemic (2015-2019)		Pandemic (2020)	
	January to March	April to December	January to March	April to December
**No. of in-hospital deaths**				
ACSC	36.1 (11.3)	28.0 (6.5)	31.0 (4.6)	24.8 (3.9)
Acute	5.9 (3.0)	5.3 (2.0)	3.7 (0.6)	5.4 (2.2)
Chronic	21.7 (7.2)	17.4 (5.0)	18.7 (0.6)	15.0 (4.4)
Vaccine-preventable	8.4 (3.5)	5.3 (2.9)	8.7 (4.2)	4.3 (2.4)
AMI	5.9 (2.9)	4.7 (2.2)	7.0 (3.5)	3.7 (1.2)
**No. of deaths within 24 h of hospital arrival**				
ACSC	5.1 (2.1)	3.6 (1.9)	5.0 (1.0)	5.4 (3.6)
Acute	0.7 (0.6)	0.4 (0.7)	0	1.0 (1.0)
Chronic	3.5 (1.3)	2.7 (1.7)	4.3 (1.2)	3.6 (2.7)
Vaccine-preventable	0.8 (1.1)	0.5 (0.8)	0.7 (0.6)	0.9 (1.2)
AMI	3.1 (2.2)	2.5 (1.7)	4.3 (2.5)	2.2 (1.2)
**No. of deaths after 24 h of hospital arrival**				
ACSC	31.0 (10.7)	24.4 (5.9)	26.0 (3.6)	19.3 (5.5)
Acute	5.2 (2.7)	4.9 (1.9)	3.7 (0.6)	4.4 (1.7)
Chronic	18.2 (6.9)	14.8 (4.3)	14.3 (1.5)	11.4 (4.9)
Vaccine-preventable	7.6 (3.5)	4.7 (2.7)	8.0 (4.4)	3.4 (2.5)
AMI	2.8 (1.4)	2.2 (1.4)	2.7 (1.5)	1.4 (1.3)
**No. of hospitalizations**				
ACSC	435.7 (54.2)	393.9 (35.5)	413.3 (69.9)	313.3 (38.1)
Acute	92.5 (14.6)	107.2 (17.6)	84.0 (10.2)	93.0 (28.9)
Chronic	261.7 (32.3)	235.2 (30.1)	255.0 (29.3)	193.2 (23.7)
Vaccine-preventable	81.6 (27.5)	51.4 (10.9)	74.3 (33.5)	27.1 (6.0)
AMI	46.3 (9.0)	41.9 (7.7)	46.0 (7.2)	37.3 (6.9)
**In-hospital mortality rate** ^a^				
ACSC	0.082 (0.018)	0.071 (0.015)	0.075 (0.009)	0.080 (0.017)
Acute	0.065 (0.035)	0.050 (0.020)	0.044 (0.010)	0.058 (0.016)
Chronic	0.082 (0.019)	0.074 (0.017)	0.074 (0.008)	0.077 (0.018)
Vaccine-preventable	0.103 (0.033)	0.100 (0.049)	0.120 (0.054)	0.176 (0.120)
AMI	0.126 (0.062)	0.111 (0.049)	0.147 (0.049)	0.103 (0.042)
**In-hospital mortality rate within 24 h of hospital arrival** ^a^				
ACSC	0.012 (0.004)	0.009 (0.005)	0.012 (0.001)	0.018 (0.015)
Acute	0.008 (0.006)	0.004 (0.006)	0	0.011 (0.010)
Chronic	0.014 (0.005)	0.011 (0.007)	0.017 (0.004)	0.019 (0.016)
Vaccine-preventable	0.010 (0.013)	0.010 (0.014)	0.009 (0.009)	0.043 (0.071)
AMI	0.066 (0.054)	0.059 (0.038)	0.090 (0.040)	0.062 (0.038)

Abbreviations: ACSC, ambulatory care–sensitive condition; AMI, acute myocardial infarction.

^a^ The in-hospital mortality rate was defined as the number of deaths divided by the number of hospitalizations per month. The in-hospital mortality rate within 24 hours was defined as the number of deaths within 24 hours of hospital arrival divided by the number of hospitalizations per month.

In Table 2, DID with Poisson regression showed that the number of hospitalizations decreased overall (IRR, 0.84; 95% CI, 0.75-0.94), for chronic conditions (IRR, 0.84; 95% CI, 0.77-0.92), and for vaccine-preventable conditions (IRR, 0.58; 95% CI, 0.44-0.76). Furthermore, the numbers of in-hospital deaths (IRR, 1.66; 95% CI, 1.15-2.39) and deaths within 24 hours of hospital arrival (IRR, 7.27 × 10^6^; 95% CI, 1.83 × 10^6^ to 2.89 × 10^7^) for acute conditions were increased. The major contributory factors in the increase in in-hospital deaths within 24 hours (April-December of prepandemic vs pandemic periods) were dehydration and gastroenteritis (eFigure 1 in Supplement 1). Additionally, there was a slight increase in the percentage of bacterial pneumonia.

**Table 2.** IRRs Estimated by the Difference-in-Difference Approach^a^

Outcome variable	IRR (95% CI)				
	ACSC category				AMI
	Total	Acute	Chronic	Vaccine-preventable	
No. of in-hospital deaths	0.93 (0.82 to 1.04)	1.66 (1.15 to 2.39)	1.00 (0.75 to 1.33)	0.80 (0.53 to 1.19)	0.66 (0.44 to 0.98)
Within 24 h of hospital arrival	1.04 (0.93 to 1.16)	7.27 × 10^6^ (1.83 × 10^6^ to 2.89 × 10^7^)	1.09 (0.71 to 1.66)	2.00 (0.55 to 7.31)	0.63 (0.39 to 1.01)
After 24 h of hospital arrival	0.88 (0.76 to 1.03)	1.30 (0.89 to 1.88)	0.98 (0.67 to 1.45)	0.69 (0.42 to 1.13)	0.70 (0.33 to 1.51)
No. of hospitalizations	0.84 (0.75 to 0.94)	0.95 (0.81 to 1.13)	0.84 (0.77 to 0.92)	0.58 (0.44 to 0.76)	0.90 (0.73 to 1.10)
In-hospital mortality rates^b^	1.22 (0.96 to 1.56)	1.71 (1.16 to 2.54)	1.15 (0.87 to 1.53)	1.51 (0.92 to 2.49)	0.65 (0.44 to 0.97)
Within 24 h of hospital arrival^b^	1.87 (1.19 to 2.96)	2.15 × 10^6^ (5.25 × 10^5^ to 8.79 × 10^6^)	1.33 (0.84 to 2.12)	4.64 (1.28 to 16.77)	0.77 (0.46 to 1.30)

Abbreviations: ACSC, ambulatory care–sensitive condition; AMI, acute myocardial infarction; IRR, incidence rate ratio.

^a^ Poisson regression analysis was performed using robust estimators for the intersection term of the dummy variable for prepandemic and pandemic periods as well as the dummy variable for before and after April, along with their respective first-order terms.

^b^ The in-hospital mortality rate was defined as the number of deaths divided by the number of hospitalizations per month. The 24-hour in-hospital mortality rate was defined as the number of deaths within 24 hours of hospital arrival divided by the number of hospitalizations per month.

The in-hospital mortality rate increased for acute conditions (IRR, 1.71; 95% CI, 1.16-2.54), and the 24-hour in-hospital mortality rates also increased overall (IRR, 1.87; 95% CI, 1.19-2.96), for acute conditions (IRR, 2.15 × 10^6^; 95% CI, 5.25 × 10^5^ to 8.79 × 10^6^), and for vaccine-preventable conditions (IRR, 4.64; 95% CI, 1.28-16.77). The same trend was observed after adjusting for age, sex, and comorbidity index (eTable 3 in Supplement 1). By comparison, all outcomes for AMI showed a decreasing trend.

The length of stay for ACSC overall decreased after the national state of emergency was declared (IRR, 0.87; 95% CI, 0.76-0.98). In addition, ambulance transport rates increased overall (IRR, 1.10; 95% CI, 1.03-1.17) and for acute conditions (IRR, 1.22; 95% CI, 1.05-1.42) (eTable 4 in Supplement 1).

In the triple-difference estimation, hospitals with more than 200 beds had a smaller increase in the number of in-hospital deaths within 24 hours of hospital arrival (IRR, 2.27 × 10^–7^; 95% CI, 3.03 × 10^–7^ to 1.71 × 10^–5^) and mortality rate within 24 hours of hospital arrival (IRR, 1.40 × 10^–6^; 95% CI, 1.76 × 10^–7^ to 1.12 × 10^–5^) compared with smaller hospitals (Table 3; eTable 5 in Supplement 1). Monthly IRRs for each outcome were bimodal or trimodal in shape (eFigure 2 in Supplement 1).

**Table 3.** IRRs Estimated by the Triple-Difference Approach^a^

Outcome variable	ACSC category, IRR (95% CI)			
	Total	Acute	Chronic	Vaccine-preventable
No. of in-hospital deaths	0.73 (0.26 to 2.05)	1.43 (0.15 to 13.48)	0.62 (0.19 to 2.03)	0.53 (0.12 to 2.30)
Within 24 h of hospital arrival	2.27 × 10^–7^ (3.03 × 10^–7^ to 1.71 × 10^–5^)	1.31 × 10^–6^ (1.20 × 10^–7^ to 1.42 × 10^–5^)	4.03 × 10^–6^ (2.73 × 10^–7^ to 5.95 × 10^–5^)	7.52 × 10^–7^ (2.31 × 10^–8^ to 2.45 × 10^–5^)
After 24 h of hospital arrival	0.92 (0.33 to 2.54)	4.00 (0.30 to 53.85)	0.67 (0.20 to 2.20)	0.74 (0.13 to 4.23)
No. of hospitalizations	0.92 (0.60 to 1.41)	0.96 (0.46 to 2.00)	0.96 (0.65 to 1.42)	0.78 (0.31 to 2.01)
In-hospital mortality rate^b^	0.67 (0.24 to 1.90)	3.85 (0.40 to 37.49)	0.59 (0.20 to 1.72)	0.53 (0.11 to 2.61)
Within 24 h of hospital arrival^b^	1.40 × 10^–6^ (1.76 × 10^–7^ to 1.12 × 10^–5^)	1.35 × 10^–7^ (7.75 × 10^–9^ to 2.37 × 10^–6^)	1.30 × 10^–5^ (8.55 × 10^–7^ to 1.97 × 10^–4^)	1.33 × 10^–7^ (3.96 × 10^–9^ to 4.44 × 10^–6^)

Abbreviations: ACSC, ambulatory care–sensitive condition; IRR, incidence rate ratio.

^a^ Poisson regression analysis was performed using robust estimators for the third-order intersection terms of the dummy variable for the prepandemic and pandemic periods, the dummy variable for before March or after April, and the dummy variable for more or fewer than 200 beds, along with each first-order and second-order intersection term.

^b^ The in-hospital mortality rate was defined as the number of deaths divided by the number of hospitalizations per month. The in-hospital mortality rate within 24 hours was defined as the number of deaths within 24 hours of hospital arrival divided by the number of hospitalizations per month.

## Discussion

In this cohort study, we used a DID approach to examine changes in the number of in-hospital deaths and mortality rates among patients with ACSC in the prepandemic vs pandemic periods. We found that in-hospital mortality rates within 24 hours of hospital arrival increased overall by 87% (IRR, 1.87) as well as for acute and vaccine-preventable conditions. The increased mortality rates were associated with not only a decrease in hospitalizations (denominator) but also an increase in in-hospital deaths (numerator). The proportions of dehydration and gastroenteritis as well as bacterial pneumonia increased as contributory factors in the death within 24 hours of hospital arrival of patients with ACSC. Furthermore, the increase in 24-hour in-hospital mortality was lower in larger hospitals (>200 beds). Length of stay for patients with ACSC decreased in the pandemic period by 13% (IRR, 0.87), whereas ambulance transport rates increased by 10% (IRR, 1.10), especially for acute conditions.

A decrease in the number of hospitalizations and emergency department visits for ACSC during the early stages of the COVID-19 pandemic was also reported in previous studies conducted in Canada, Japan, and the US, despite variation in COVID-19 rates.^14,15,17^ While some previous studies conducted regression analysis based on a before-vs-after comparison, adjusted for covariates, the present analysis adopted a DID design to address possible unmeasured confounders such as socioeconomic status. A new finding in this study was that, despite the decrease in the number of hospitalizations, the number of deaths and in-hospital mortality rates increased during the pandemic, especially for patients with acute ACSC.

Several mechanisms could explain the increased number of deaths associated with acute ACSC. First, patients with acute ACSC might not have had access to high-quality outpatient care during the pandemic.^25^ According to the definition of ACSC, hospitalization of patients with ACSC may be preventable to some extent through outpatient care.^12^ Nevertheless, the increase in the number of in-hospital deaths and in-hospital deaths within 24 hours of hospital arrival among patients with acute ACSC, as well as the increased rate of ambulance transport to hospitals, suggest problems with access to and/or quality of primary care during the pandemic. Under prepandemic conditions in Japan, patients with fever or catarrhal symptoms were able to visit almost any outpatient clinic with minor cost-sharing and without an appointment.^21^ However, during the pandemic, patients with fever or catarrhal symptoms were able to be seen only at designated outpatient clinics that were recommended by the local public health center. The reason for this restriction was that the Japanese government sought to optimize limited polymerase chain reaction (PCR) testing capacity and to conduct epidemiological surveys.^16,26^ The sheer number of febrile patients made it difficult to connect to a public health center’s telephone lines, and access to PCR testing was further challenged by the limited number of designated clinics and by the stringent criteria for PCR testing eligibility (eg, history of intense contact with confirmed cases of COVID-19, fever and dyspnea, or travel to an endemic area within the past 2 weeks).^16,27,28^ Following the government guidelines, many medical institutions turned away febrile patients and instructed them to consult with their public health centers first. In addition, many patients may have been reluctant to seek care in hospitals for fear of contracting COVID-19 infection.^1,2^ As a result, a prior study reported a decrease in the number of outpatient visits after the declaration of the national state of emergency.^29^ Thus, many patients with COVID-19-like symptoms stayed home without undergoing PCR testing or accessing primary care. Reduced access to primary care for patients with acute ACSC may have contributed to their subsequent deaths.

Even if ACSC-related hospitalizations were considered to be preventable in the outpatient setting, some degree of inpatient care is necessary. Hence, a second explanation for the findings could be the difficulties experienced by patients with acute ACSC in accessing inpatient care. For example, a study reported lengthy delays in ambulances finding a hospital that would accept patients during the pandemic.^30,31^ Moreover, since resources for acute care were often concentrated in hospitals with 200 or more beds in Japan, these few larger hospitals were overwhelmed with patients with COVID-19.^31^ For smaller hospitals, the greater increase in the number of in-hospital deaths within 24 hours of hospital arrival implied that some patients should have been diverted to larger hospitals.

The statistically significant increase in the number of in-hospital deaths for acute ACSC, even after adjusting for patient age, sex, and comorbidity index, may be indicative of a decline in the quality of in-hospital care. Although the in-hospital mortality rate for AMI did not worsen in this study, and patient outcomes for AMI and emergency abdominal surgery were reported in previous studies to have remained unchanged during the pandemic,^22,32^ the quality of in-hospital care for COVID-19-like symptoms may have declined.

### Limitations

This study has some limitations. First, the DID approach was based on the parallel trend assumption and the common shock assumption.^18^ We demonstrated parallel trends for most outcomes for the months preceding the COVID-19 national state of emergency declaration in Japan (eFigure 2 in Supplement 1). As for the common shock assumption, a sensitivity analysis was conducted to account for potential confounders, yet the observed pattern remained unchanged. Further studies are needed to determine the implications of the pandemic for patient heterogeneity. Second, this study used the ACSC definition used in the UK National Health Service but has not yet been validated in Japan. Still, several previous studies in Japan have used it,^17,21^ and thus the findings of the present study are comparable to the findings of the past reports. Third, patients with ACSC may have been admitted with COVID-19 (*ICD-10* code U07.1). In Japan, PCR testing was performed on all patients who were admitted to the hospital during the pandemic, but we cannot rule out false-negative results. However, as far as we can recognize from the data, there were no ACSC-related hospitalizations that listed COVID-19 (*ICD-10* code U07.1) as a comorbidity at admission or as a new condition developing during hospitalization. Fourth, the cause of death was not clear in these data. Therefore, the admission diagnosis itself may not have caused the deaths. Fifth, due to the small number of monthly ACSC-related in-hospital deaths (especially deaths within 24 hours of hospital arrival), some IRRs had extreme values in the regression analysis. Further analysis with larger sample size data is needed. Sixth, the results of this study cannot be directly generalized to other countries because the extent of and response to the COVID-19 pandemic differed among countries. However, the results could shed light on whether patients presenting with COVID-19-like symptoms received appropriate primary care and in-hospital care during the pandemic.

## Conclusions

This cohort study found that, during the COVID-19 pandemic in Japan, the number of in-hospital deaths increased, particularly for patients with acute ACSC and within 24 hours of hospital arrival. This finding suggests that access to good-quality primary care and inpatient care for patients with COVID-19-like symptoms could have been compromised during the pandemic. In an infectious disease–related public health crisis, planning is needed to ensure continued access to medical care for patients with similar symptomatic illnesses.

## Notes

Supplement 1. **eFigure 1.** Changes in the Proportion of Diagnosis Among In-Hospital Deaths Within 24 Hours of Hospital Arrival From April to December for Pre-pandemic and Pandemic Periods **eFigure 2.** Trends of Monthly Incidence-Rate Ratios of Each Outcome in ACSC Hospitalizations Estimated by the Difference in Differences **eTable 1.** Ambulatory Care Sensitive Conditions (ACSC) and ICD-10 Codes **eTable 2.** Monthly Statistics of Covariates and Secondary Outcomes of ACSC Hospitalizations in Pre-pandemic and Pandemic Periods **eTable 3.** Adjusted Incidence-Rate Ratios (95% Confidence Intervals) Estimated by the Difference-in-Difference Approach **eTable 4.** Incidence-Rate Ratios (95% Confidence Intervals) of Secondary Outcomes, Estimated by the Difference-in-Difference Approach **eTable 5.** Adjusted Incidence-Rate Ratios (95% Confidence Intervals) Estimated by Triple Difference Approach

Supplement 2.
Data Sharing Statement
