# Supplementary material for: In-Hospital Deaths From Ambulatory Care–Sensitive Conditions Before and During the COVID-19 Pandemic in Japan
Source: JAMA Netw Open. 2023 Jun 22;6(6):e2319583. doi: 10.1001/jamanetworkopen.2023.19583 (PMC10288336; doi:10.1001/jamanetworkopen.2023.19583)
Supplement: Supplement 1. — eFigure 1. Changes in the Proportion of Diagnosis Among In-Hospital Deaths Within 24 Hours of Hospital Arrival From April to December for Pre-pandemic and Pandemic Periods eFigure 2. Trends of Monthly Incidence-Rate Ratios of Each Outcome in ACSC Hospitalizations Estimated by the Difference in Differences eTable 1. Ambulatory Care Sensitive Conditions (ACSC) and ICD-10 Codes eTable 2. Monthly Statistics of Covariates and Secondary Outcomes of ACSC Hospitalizations in Pre-pandemic and Pandemic Periods eTable 3. Adjusted Incidence-Rate Ratios (95% Confidence Intervals) Estimated by the Difference-in-Difference Approach eTable 4. Incidence-Rate Ratios (95% Confidence Intervals) of Secondary Outcomes, Estimated by the Difference-in-Difference Approach eTable 5. Adjusted Incidence-Rate Ratios (95% Confidence Intervals) Estimated by Triple Difference Approach [file jamanetwopen-e2319583-s001.pdf]

Supplemental Online Content

Abe K, Kawachi I, Iba A, Miyawaki A. In-hospital deaths from ambulatory care–sensitive conditions before and during the COVID-19 pandemic in Japan. *JAMA Netw Open*. 2023;6(6):e2319583. doi:10.1001/jamanetworkopen.2023.19583

eFigure 1. Changes in the Proportion of Diagnosis Among In-Hospital Deaths Within 24 Hours of Hospital Arrival From April to December for Pre-pandemic and Pandemic Periods

eFigure 2. Trends of Monthly Incidence-Rate Ratios of Each Outcome in ACSC Hospitalizations Estimated by the Difference in Differences

eTable 1. Ambulatory Care Sensitive Conditions (ACSC) and ICD-10 Codes

eTable 2. Monthly Statistics of Covariates and Secondary Outcomes of ACSC Hospitalizations in Pre-pandemic and Pandemic Periods

eTable 3. Adjusted Incidence-Rate Ratios (95% Confidence Intervals) Estimated by the Difference-in-Difference Approach

eTable 4. Incidence-Rate Ratios (95% Confidence Intervals) of Secondary Outcomes, Estimated by the Difference-in-Difference Approach

eTable 5. Adjusted Incidence-Rate Ratios (95% Confidence Intervals) Estimated by Triple Difference Approach

This supplemental material has been provided by the authors to give readers additional information about their work.

© 2023 Abe K et al. *JAMA Network Open.*

eFigure 1. Changes in the proportion of diagnosis among in-hospital deaths within 24 hours of hospital arrival from April to December for pre-pandemic and pandemic periods

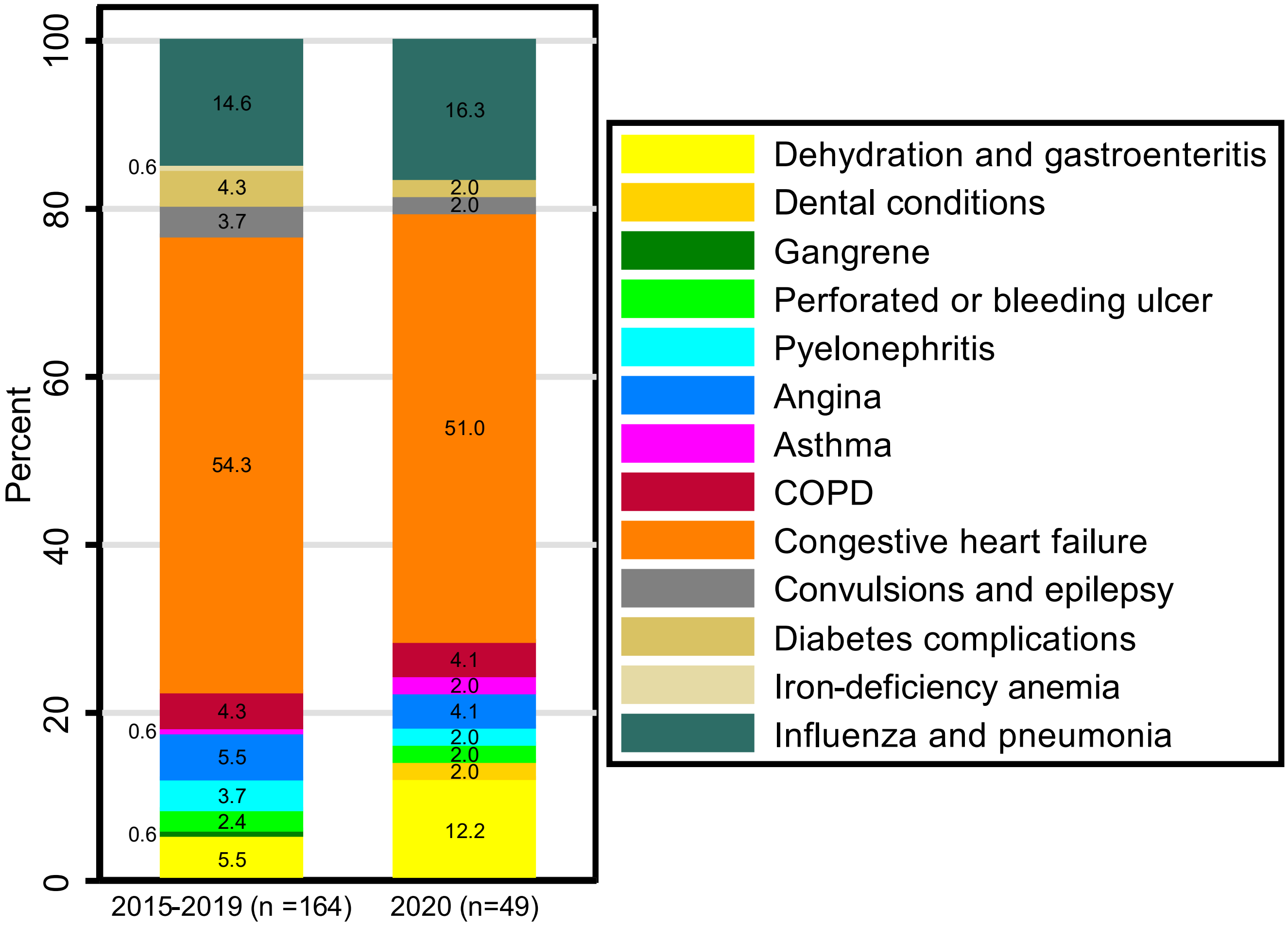

COPD indicates chronic obstructive pulmonary disease. Diagnosis of deaths in which there were no deaths in both periods was not included.

© 2023 Abe K et al. *JAMA Network Open.*

eFigure 2. Trends of monthly incidence-rate ratios of each outcome in ACSC hospitalizations estimated by the difference in differences

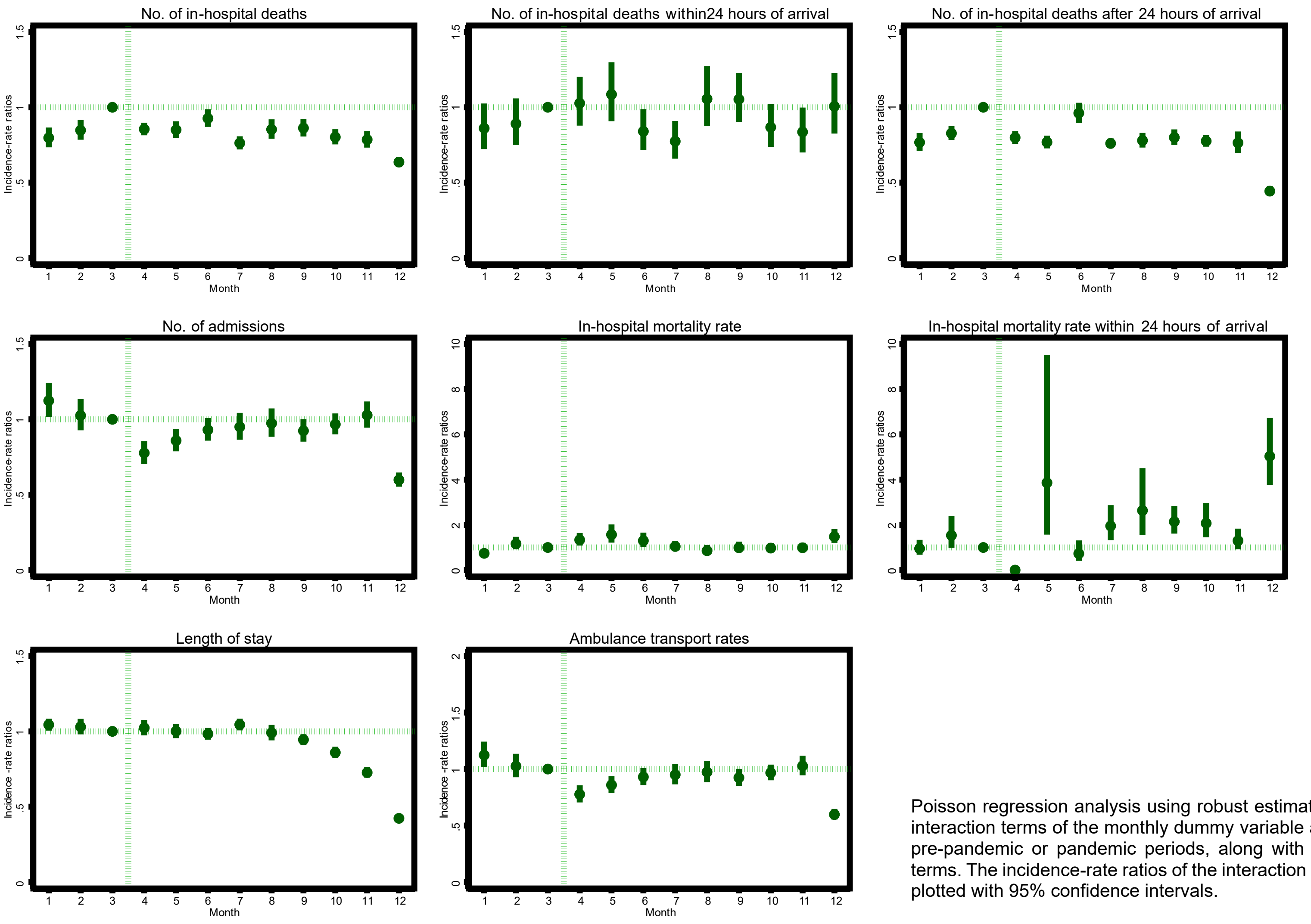

Poisson regression analysis using robust estimators was performed for the interaction terms of the monthly dummy variable and the dummy variable of pre-pandemic or pandemic periods, along with their respective first-order terms. The incidence-rate ratios of the interaction terms for each month were plotted with 95% confidence intervals.

© 2023 Abe K et al. *JAMA Network Open.*

eTable 1. Ambulatory care sensitive conditions (ACSC) and ICD-10 codes

ACSC category	ACSC	ICD-10 code
**Acute**	**Cellulitis**	L03, L04, L08.0, L08.8, L08.9, L88, L98.0
	**Dehydration and gastroenteritis**	E86, K52.2, K52.8, K52.9
	**Dental conditions**	A69.0, K02, K03, K04, K05, K06, K08, K09.8, K09.9, K12, K13
	**Ear, nose, and throat infections**	H66, H67, J02, J03, J06, J31.2
	**Gangrene**	R02
	**Nutritional deficiency**	E40, E41, E42, E43, E55.0, E64.3
	**Pelvic inflammatory disease**	N70, N73, N74
	**Perforated or bleeding ulcer**	K25.0-K25.2, K25.4-25.6, K26.0-K26.2, K26.4-26.6, K27.0-27.2, K27.4-K27.6, K280-K282, K284-K286
	**Pyelonephritis**	N10, N11, N12, N13.6
**Chronic**	**Angina**	I20, I24.0, I24.8, I24.9
	**Asthma**	J45, J46
	**Chronic obstructive pulmonary disease**	J20, J41, J42, J43, J44, J47
	**Congestive heart failure**	I11.0, I50, J81
	**Convulsions and epilepsy**	G40, G41, R56, O15
	**Diabetes complications**	E10.0-E10.8, E11.0-E11.8, E12.0-E12.8, E13.0-E13.8, E14.0-E14.8
	**Hypertension**	I10, I11.9
	**Iron-deficiency anemia**	D50.1, D50.8, D50.9
**Vaccine-preventable**	**Influenza and pneumonia**	J10, J11, J13, J14, J15.3, J15.4, J15.7, J15.9, J16.8, J18.1, J18.8
	**Other vaccine-preventable diseases**	A35, A36, A37, A80, B05, B06, B16.1, B16.9, B18.0, B18.1, B26, G00.0, M01.4

© 2023 Abe K et al. *JAMA Network Open.*

eTable 2. Monthly statistics of covariates and secondary outcomes of ACSC[a] hospitalizations in pre-pandemic and pandemic periods

	Pre-pandemic term (2015-2019)		Pandemic term (2020)	
	January to March, mean (SD)	April to December, mean (SD)	January to March, mean (SD)	April to December, mean (SD)
**Age, median (IQR)**				
**ACSC**	77.0 (75.0-78.0)	75.0 (74.0-76.0)	77.0 (77.0-79.0)	77.5 (77.0-79.0)
**Acute**	73.0 (72.0-74.0)	74.0 (72.0-75.0)	76.0 (75.0-76.0)	76.5 (75.5-77.0)
**Chronic**	77.0 (76.0-78.0)	74.0 (73.0-76.0)	79.0 (77.0-79.0)	78.0 (77.0-78.0)
**Vaccine-preventable**	79.0 (76.0-81.0)	78.0 (76.0-80.5)	79.0 (76.0-83.0)	80.0 (77.0-82.5)
**Acute myocardial infarction**	71.0 (68.5-73.0)	71.0 (69.0-74.0)	70.5 (70.0-74.0)	72.0 (71.0-73.0)
**Proportion of women, % (SD)**				
**ACSC**	46.4 (3.1)	45.9 (2.6)	44.6 (1.7)	45.1 (3.3)
**Acute**	52.7 (6.1)	51.2 (4.6)	46.1 (5.8)	55.5 (2.9)
**Chronic**	45.3 (3.7)	44.7 (3.2)	44.9 (2.6)	41.6 (4.6)
**Vaccine-preventable**	42.0 (4.5)	40.7 (6.7)	41.9 (14.5)	36.1 (9.5)
**Acute myocardial infarction**	28.9 (8.3)	26.4 (7.1)	25.1 (2.6)	27.1 (7.7)
**Elixhauser comorbidity index**				
**ACSC**	1.43 (0.06)	1.37 (0.08)	1.47 (0.06)	1.49 (0.11)
**Acute**	1.12 (0.08)	1.18 (0.11)	1.16 (0.19)	1.19 (0.16)
**Chronic**	1.58 (0.06)	1.45 (0.10)	1.61 (0.06)	1.62 (0.11)
**Vaccine-preventable**	1.34 (0.08)	1.36 (0.16)	1.32 (0.11)	1.45 (0.22)
**Acute myocardial infarction**	1.63 (0.16)	1.61 (0.20)	1.57 (0.20)	1.52 (0.16)
**Length of stay**				
**ACSC**	17.6 (1.4)	16.4 (0.9)	17.6 (0.7)	15.8 (3.3)
**Acute**	16.5 (3.5)	15.3 (1.9)	14.8 (2.2)	14.5 (3.7)
**Chronic**	17.8 (1.3)	16.6 (1.3)	18.2 (1.2)	16.0 (3.3)
**Vaccine-preventable**	18.2 (3.3)	17.5 (2.7)	19.2 (4.9)	18.3 (5.6)
**Acute myocardial infarction**	14.7 (2.0)	14.8 (2.0)	12.8 (2.1)	13.9 (2.6)
**Ambulance transport rate[b]**				
**ACSC**	0.458 (0.035)	0.420 (0.028)	0.486 (0.013)	0.488 (0.032)
**Acute**	0.354 (0.054)	0.356 (0.044)	0.352 (0.037)	0.433 (0.044)
**Chronic**	0.495 (0.033)	0.459 (0.039)	0.535 (0.012)	0.517 (0.040)
**Vaccine-preventable**	0.452 (0.074)	0.373 (0.088)	0.472 (0.056)	0.452 (0.087)
**Acute myocardial infarction**	0.685 (0.059)	0.718 (0.073)	0.733 (0.015)	0.725 (0.062)

a. ACSC means ambulatory care sensitive conditions.
b. The ambulance transport rate is the ratio of ambulance transports to the number of hospitalizations.

© 2023 Abe K et al. *JAMA Network Open.*

eTable 3. Adjusted incidence-rate ratios (95% Confidence Intervals) estimated by the difference-in-difference approach[a]

	Ambulatory care sensitive conditions				Acute myocardial infarction
	Total	Acute	Chronic	Vaccine-preventable	
**No. of in-hospital deaths**	0.93 (0.82 - 1.04)	2.00 (1.37 - 2.91)	0.90 (0.68 - 1.20)	0.74 (0.47 - 1.17)	0.65 (0.44 - 0.97)
**Within 24 hours[b]**	1.04 (0.91 - 1.18)	1.93e+7 (4.05e+6 – 9.10e+7)	1.03 (0.68 - 1.56)	1.78 (0.42 - 7.43)	0.67 (0.40 - 1.13)
**After 24 hours[c]**	0.89 (0.77 - 1.03)	1.55 (1.05 - 2.30)	0.86 (0.59 - 1.25)	0.64 (0.37 - 1.11)	0.67 (0.33 - 1.34)
**No. of hospitalizations**	0.82 (0.75 - 0.89)	0.90 (0.77 - 1.06)	0.82 (0.76 - 0.89)	0.59 (0.45 - 0.78)	0.96 (0.76 - 1.22)
**In-hospital mortality rates[d]**	1.22 (0.99 - 1.51)	2.04 (1.39 - 3.00)	1.10 (0.83 - 1.45)	1.47 (1.02 - 2.12)	0.75 (0.49 - 1.14)
**Within 24 hours[d]**	1.70 (1.05 - 2.77)	2.15e+5 (5.25e+5 – 8.79e+6)	1.34 (0.85 - 2.11)	3.88 (1.00 - 15.09)	0.80 (0.44 - 1.47)
**Length of stay**	0.90 (0.82 - 0.98)	1.05 (0.89 - 1.25)	0.86 (0.75 - 0.98)	0.98 (0.73 - 1.33)	1.08 (0.95 - 1.24)
**Ambulance transport rate[e]**	1.06 (1.00 - 1.12)	1.16 (1.00 - 1.35)	1.00 (0.94 - 1.08)	1.07 (0.90 - 1.28)	0.93 (0.86 - 1.01)

a. Poisson regression analysis was performed using robust estimators for the intersection term of the dummy variable for pre-pandemic and pandemic periods, and the dummy variable for before and after April, along with their respective first-order terms. Patients' age, sex, and Elixhouser comorbidity index at admission were adjusted.
b. The number of in-hospital deaths within 24 hours of arrival at the hospital.
c. The number of in-hospital deaths after 24 hours of arrival at the hospital.
d. The in-hospital mortality rate was defined as the number of deaths divided by the number of hospitalizations per month; the 24-hour in-hospital mortality rate was defined as the number of deaths within 24 hours of hospital arrival divided by the number of hospitalizations per month.
e. The ambulance transport rate is the ratio of ambulance transports to the number of hospitalizations.

© 2023 Abe K et al. *JAMA Network Open.*

eTable 4. Incidence-rate ratios (95% Confidence Intervals) of secondary outcomes, estimated by the difference-in-difference approach[a]

	Ambulatory care sensitive conditions				Acute myocardial infarction
	Total	Acute	Chronic	Vaccine-preventable	
**Length of stay**	0.87 (0.76-0.98)	1.06 (0.86-1.31)	0.94 (0.83-1.06)	0.99 (0.75-1.32)	1.08 (0.89-1.32)
**Ambulance transport rate[b]**	1.10 (1.03-1.17)	1.22 (1.05-1.42)	1.04 (0.98-1.11)	1.16 (0.93-1.45)	0.94 (0.87-1.02)

a. Poisson regression analysis was performed using robust estimators for the intersection term of the dummy variable for pre-pandemic and pandemic terms, and the dummy variable for before and after April, along with their respective first-order terms.

b. The ambulance transport rate is the ratio of ambulance transports to the number of hospitalizations.

eTable 5. Adjusted incidence-rate ratios (95% Confidence Intervals) estimated by Triple difference approach[a]

	Ambulatory care sensitive conditions			
	**Total**	**Acute**	**Chronic**	**Vaccine-preventable**
**No. of in-hospital deaths**	0.99<br>(0.43 - 2.31)	1.72<br>(0.19 - 15.30)	0.80<br>(0.25 - 2.61)	0.77<br>(0.20 - 3.01)
**Within 24 hours**	7.04e-6<br>(8.71e-7 - 5.69e-5)	4.19e-7<br>(3.49e-8 - 5.03e-6)	2.90e-5<br>(1.54e-6 - 5.46e-4)	9.05e-6<br>(1.76e-7 - 4.67e-4)
**After 24 hours**	1.18<br>(0.51 - 2.69)	4.87<br>(0.38 - 61.99)	0.79<br>(0.25 - 2.49)	0.92<br>(0.16 - 5.21)
**No. of hospitalizations**	0.82<br>(0.52 - 1.29)	0.83<br>(0.38 - 1.83)	0.89<br>(0.59 - 1.34)	0.61<br>(0.23 - 1.62)
**In-hospital mortality rate[b]**	0.85<br>(0.36 - 2.01)	4.73<br>(0.48 - 47.06)	0.76<br>(0.26 - 2.21)	0.72<br>(0.21 - 2.49)
**Within 24 hours[b]**	6.86e-6<br>(8.59e-7 - 5.48e-5)	9.11e-08<br>(3.66e-09 - 2.26e-06)	7.60e-5<br>(3.56e-6 - 1.62e-3)	3.60e-7<br>(1.55e-9 - 8.39e-5)

a. Poisson regression analysis was performed using robust estimators for the third-order intersection terms of the dummy variable for pre- and pandemic terms, the dummy variable for before March or after April, and the dummy variable for more or less than 200 beds, along with each first-order term and second-order intersection term. Patients' age, sex, and Elixhauser comorbidity index at admission were adjusted.

b. The in-hospital mortality rate was defined as the number of deaths divided by the number of hospitalizations per month; the in-hospital mortality rate within 24 hours was defined as the number of deaths within 24 hours of hospital arrival divided by the number of hospitalizations per month.

© 2023 Abe K et al. *JAMA Network Open.*
