# Supplementary material for: In-Hospital Deaths From Ambulatory Care–Sensitive Conditions Before and During the COVID-19 Pandemic in Japan
Source: JAMA Netw Open. 2023 Jun 22;6(6):e2319583. doi: 10.1001/jamanetworkopen.2023.19583 (PMC10288336; doi:10.1001/jamanetworkopen.2023.19583)
Supplement: Supplement 2. — Data Sharing Statement [file jamanetwopen-e2319583-s002.pdf]

Data Sharing Statement

Abe. In-Hospital Deaths From Ambulatory Care–Sensitive Conditions Before and During the COVID-19 Pandemic in Japan. *JAMA Netw Open*. Published June 22, 2023. doi:10.1001/jamanetworkopen.2023.19583

Data
Data available: No

Additional Information
Explanation for why data not available: Data may be obtained from a third party and are not publicly available. We obtained the data from the Medical Data Vision Co.(MDV), and we are not allowed to share these data with other parties. However, researchers who meet the criteria for access can acquire de-identified participant data from the MDV (https://en.mdv.co.jp).
